# Human Impairment from Living near Confined Animal (Hog) Feeding Operations

**DOI:** 10.1155/2012/565690

**Published:** 2012-02-09

**Authors:** Kaye H. Kilburn

**Affiliations:** ^1^Keck School of Medicine, University of Southern California, Los Angeles, CA 90033, USA; ^2^Neuro-Test, Inc., 3250 Mesaloa Lane, Pasadena, CA 91107, USA

## Abstract

*Problem.* To determine whether neighbors around manure lagoons and massive hog confinement buildings who complained of offensive odors and symptoms had impaired brain and lung functions. *Method.* We compared near hog manure neighbors of lagoons to people living beyond 3 kilometers in Ohio and to unexposed people controls in a nearby state for neurophysiological, cognitive, recall and memory functions, and pulmonary performance. *Results.* The 25 exposed subjects averaged 4.3 neurobehavioral abnormalities, significantly different from 2.5 for local controls and 2.3 for Tennessee controls. Exposed subjects mean forced vital capacity and expiratory volume in 1 sec were reduced significantly compared to local and regional controls. *Conclusions.* Near neighbors of hog enclosures and manure lagoon gases had impaired neurobehavioral functions and pulmonary functions and these effects extended to nearby people thought to be controls. Hydrogen sulfide must be abated because people living near lagoons cannot avoid rotten egg gas.

## 1. Introduction

Centralizing animal production has increased efficiency and reduced costs of meat and dairy products. The disadvantage is that objectionable odors emanate from huge quantities of manure that are generated daily. Confined animal feed operations (CAFOs) exposed their human neighbors to effluent gases including hydrogen sulfide, and other sulfur gases, ammonia, pork antigens, and aerosols.

Beyond being unpleasant, these gases adversely affect human lungs, brains, and other organs. More than 100 parts per million (ppm) of hydrogen sulfide is lethal [1–7]. Permanent central nervous system impairment was described 20 years ago after nonlethal “knockdown” by hydrogen sulfide. Downwind neighbors of oil refineries, desulfurization plants and a cattle hide operation showed neurobehavioral impairment for balance, color discrimination, reaction time, and verbal recall from exposures ranging from 0.1 to 25 ppm of hydrogen sulfide [8–10]. 

Many human brain functions can be measured to estimate brain performance and losses thereof. Standing balance is simple in concept, but requires integrating in the cerebellum inputs from the vestibular apparatus (the 8th cranial nerve), ascending proprioceptive impulses, motor cortical corrections, and visual monitoring of sway. Visual perception and recognition occupies over 40% of cortical function from retinal cones for color discrimination and rods for perception in dim light. Thresholds for perception are mapped for hearing, the other 8th cranial nerve function as sound thresholds, are perceived in the brain's temporal lobes. Fingertip number writing tests parietal lobe perception. Simple/complex acts that depend upon perception decision making and response and are represented by simple and choice visual reaction time are easily tested. Most tests are measured as time needed for an act. Included are reaction times, peg placement, and making trails (connection of 25 circles in numerical order or alternate numbers and letter in alphabetical order), digit symbol substitution, and problem solving as in Culture Fair or Raven's Matrices. Comparison of observed values for each test against population-based predicted values quantifies as nearly as possible, performance before and after exposure for each of the 26 tests. Expressing performance as percentage of predicted, observed divided by predicted, improves sensitivity for individuals and when averaged, for groups.

After 4 patients living near an Ohio hog manure lagoon were diagnosed with neurobehavioral and pulmonary impairment [10], they organized their nearby neighbors and those living beyond 3 kilometers for testing. These groups were compared to each other and to unexposed people in a nearby state.

## 2. Methods

### 2.1. Exposure Measurements

Several homes were sampled and H_2_S found using direct reading Jerome meter with a 3 ppb detect limit for the near exposed people. Subsequently on May 6, 2003, air in twelve homes were monitored indoors and out for hydrogen sulfide using NIOSH method 3013-k and for ammonia with NIOSH method 5347 ISE and a handheld Jerome meter by TMC Ltd., Macedonia, OH, USA. Well water samples were analyzed for hydrogen sulfide, ammonia, oil, nitrate/nitrite, and coliform bacteria. Coliform bacteria were elevated in 5 wells but E. coli were not found. H_2_S was elevated in 2 samples to 0.11 and 1.91 mg/l whereas nitrate was under 0.5 mg/l. On February 23-24, 2005, 9 homes were monitored for H_2_S using 2 Jerome 631-x meters and 10 minutes in each room and outdoors by Burgess and Niple of Columbus, OH, USA. Water was run in bathrooms or kitchens and sample reading after 10 minutes were compared to initial values, all in ppb. 

Twenty-five people who lived in proximity to hog manure lagoons near Paulding, OH, were recruited by neighborhood canvas and scheduled for testing. Twenty-two matching unexposed people living at least 3 km from the lagoons were also tested. Hydrogen sulfide exposed and unexposed subjects were invited to volunteer without regard to complaints.

Most homes were closer than 900 meters to lagoons (range from 180 to 2,180 meters). Samples were obtained after a rain storm. Durations of residential exposure were considered, as surrogate for exposure although lagoons had existed for only 4 years. Findings are described in results.

The 22 unexposed and 25 exposed subjects were tested together on April 26, 27, 2003. Their exposure status was not identified to the testing staff to avoid bias in neurobehavioral testing as done previously [7, 11–13]. The local control group participants matched the exposed group for age and gender. They were paid $30. No subject was excluded for symptoms, annoyance or their opinions about hydrogen sulfide. No unexposed subject was medically disabled.

Comparisons were made of 22 unexposed and 25 exposed subjects to 58 Tennessee controls. Although in 2003 probably no one was truly “unexposed to chemicals,” the Tennessee registered voters from two communities were without chemical contamination by historical review and onsite inquiry. Their 2.3 average brain functional abnormalities, were distributed asymmetrically (strongly skewed left) and were similar to the population studied to derive the prediction equations [12].

These 58 reference subjects (30 women and 28 men) included 28 from Spring Hill Columbia and 30 from Waverly, TN, USA. All were volunteers who were recruited from voter registration rolls, interviewed briefly to verify their freedom from workplace and home chemical exposures, and reimbursed for time and mileage. Exposed and control subjects were intermingled for testing and their exposure status was withheld from examiners.

Exposed and referent subjects questionnaires were completed after rectifying omissions recognized by computer-guided card reading [13, 14]. Frequencies of 35 common health complaints (and two questions as validity checks) were self-rated: as rare equal to 1 to daily rated 11 [14, 15]. Other inquiries included standard lupus erythematosus questions [16], a standard respiratory questionnaire [17], histories of occupational and other exposures to chemicals, pesticides and herbicides, tobacco, alcohol, and drug use (prescription and illicit), anesthetic agents, unconsciousness, head trauma, and neurological and medical histories [14].

The questionnaires and test battery were developed and standardized in previous studies of histology technicians [18], fire fighters exposed to thermolysis products of PCBs [15], a chlorinated solvent exposed population [14], people exposed to toluene rich chemical waste, those exposed to hydrogen sulfide, and groups of unexposed subjects [5, 7, 8, 12].

Alcohol and carbon monoxide (CO) in expired alveolar air were tested by expelling a big breath held for 20 seconds using specific fuel cell analyzers [8]. No alcohol levels were above 1 ppm. Most CO levels were 0 but varied to peak at 27 ppm in persons who had smoked cigarettes within 24 hours.

### 2.2. Neurophysiological Tests

Simple reaction time (SRT) and visual two-choice reaction time (CRT) were measured with a computerized instrument [19] and the fastest median of the last 7 of two groups of 20 trials was recorded for SRT and CRT. It tests the retina and optic cortex, integrative radiation to the motor cortex, and descending corticospinal tracts.

 Body balance was measured with the subject standing erect with feet together. A sound generating stylus on a head band tracked by two microphones and processed in a computer-expressed balance as mean speed of sway in cm/sec [20]. The minimal sway speed of 3 consecutive 20-second trials was counted for sway each with eyes open and eyes closed. Balance depends on ascending proprioceptive tracts, the vestibular division of the 8th nerve, cerebellum, visual integrative, and motor tracts.

Blink reflex was measured with surface electromyographic electrodes from lateral orbicularis oculi muscles bilaterally [21, 22] after tapping the right and left supraorbital notches with a light hammer which also triggered a recording computer. Its circuit is the trigeminal nerve, pons-cross over, and motor innervation via the facial nerve. Ten firings of the first wave, R-1 were averaged for each side and failures were recorded [22].

Hearing was measured in left and right ears with standard audiometers (model ML-AM Microaudiometrics, So. Daytona, FL, USA) at stepped frequencies of 500 to 8,000 Hertz and summed for each ear. It tests the auditory division of the 8th cranial nerve.

A dynamometer measured grip for cortical motor nerve and muscle function.

Color discrimination errors were measured with the desaturated Lanthony 15 hue test under constant illumination [23] and scored with Bowman's method [24]. It tests the cones of the retina and the visual cortex.

 Visual fields were tested with a computerized (Med Lab Technology, New Wales, PA, USA) automated perimeter recording to a computer which mapped the central 30° of the right and left eye fields individually by measuring perceptual thresholds to 80 light emitting diodes. Performance was the sum of scores for each eye. Visual score counted the abnormal quadrants (scotoma or other defects) for both eyes [25]. Thus, rod functions in the retina, the optic nerve, cortical radiation were evaluated.

### 2.3. Neuropsychological Tests

Immediate verbal recall was measured by stories from Wechsler's Memory Scale-revised [26] which tests the limbic system of the temporal lobes. Culture Fair (battery 2A) and vocabulary were done in groups of 8 to 12 subjects. Culture Fair tested nonverbal nonarithmetical intelligence with 4 sets of designs for similarity, difference, completion, and pattern recognition and transfer [27, 28]. It resembles Raven's progressive matrices [29]. The 46-word vocabulary test was from Jackson's [30] multidimensional aptitude battery. Digit symbol substitution from the Wechsler Adult Intelligence Scale-revised (WAIS-R) [31] tested attention and integrative capacity. Information, picture completion and similarities from the WAIS-R tested long term retention of cultural information; a frontal lobe function that is usually maintained until chemical brain damage becomes severe [32].

Time to place 25 pegs in the Lafayette slotted pegboard with the preferred hand was measured and trail making A and B measured dexterity (optic to motor cortex), coordination and decision making. Fingertip number writing assessed peripheral sensation and discrimination were from the Halstead-Reitan battery [33, 34].

 Subjects' moods were appraised by responses to 65 terms describing emotional status for the past week using the Profile of Mood States (POMSs) [35]. It assays feeling states and the limbic system.

### 2.4. Respiratory Flows and Vital Capacities

were measured after subjects took a full inspiration and exhaled into a volume displacement (Ohio) spirometer while standing and using a nose clip and repeated until two forced expirations agreed within 5% following ATS [36] criteria. Records were traced with a digitizer, measured by a computer, compared to predicted values that adjusted for height, sex, age, and the volume and flow reducing effects of cigarette smoking, and expressed as percent of predicted [17, 37].

### 2.5. Statistical Analysis

Scores and computed data were transferred to a computer for analysis using Stata Statistical Software Version 8 (Stata Corporation, College Station, TX, USA). Without neurobehavioral testing before people were exposed to hydrogen sulfide the reasonable alternative was to calculate expected values. The combined “unexposed” population was from Tennessee [13]. The steps were the following.

1. Expected values were calculated for each test for each person using regression equations [12].
2. Expected values were based on testing unexposed general population groups with appropriate age distributions. (Test scores were mathematically transformed when this improved the symmetry of data distributions.)
3. Coefficients were retained for age for most tests, sex for many and educational attainment for problem solving, recall, long-term memory, and perceptual motor tests. Distances of homes to the nearest manure lagoon and hydrogen sulfide levels indoors were tested for influence on abnormalities score and individual test scores. Family income, hours of general anesthesia, weight, and Mood States (POMSs) scores did not influence any test.
4. Observed scores were divided by expected (predicted) scores and multiplied by 100 and expressed as percent predicted. This procedure compared each test or function to that person's calculated value that approximated baseline measurements.
5. The differences in means as percent predicted for groups were tested for statistical significance by analysis of variance.
6. Exposed groups' averaged total abnormalities were compared to averages for control groups by analysis of variance.
7. *P* values were adjusted for simultaneous inference using Holm's modification of Bonferroni procedure [38].

Each participant's total abnormality score was the sum of tests outside the 95% confidence interval (variance 92% to 97%) which was 1.5 times each test's standard deviation of each test. Balance and vision were so important in detecting effects of chemical exposure in several thousand subjects [9, 22], that each sway measures was scored 2, and visual fields performance was scored 1 for each eye. Bilateral hearing, blink reflex latency, grip strength, and fingertip number errors were assigned 0.5 per side with 1 for other functions. Regression analyses examined the effects of mood states scores, symptom frequencies, specific exposures, and other factors such as distance of their home from the hog confinement buildings.

## 3. Results

### 3.1. Exposures

Indoor air of 12 homes had hydrogen sulfide levels of 0 to 2,100 ppb. Ranges indoor and outdoor varied 10-fold or more in one-day's spot check samples. Two outdoor samples were above 1,100 ppb. Water running increased the H_2_S levels 2 to 10 times for a peak of 430 ppb.

Distances to homes from lagoons varied from 170 to 3,000 meters, the inverse of distance squared from hog confinement lagoons did not predict scores or number of abnormalities. Exposure was less than 4 years in only two people.

### 3.2. Neurobehavioral Testing

Comparison of test means for the 25 near exposed people to those of the local for exposed group of 22 showed statistically significant differences (ssd) for balance with eyes open was 1.0 abnormality, digit symbol substitution 1.0 abnormality, and vocabulary 1.0 abnormality (Table 1). These abnormalities were not statistically significant after Holm's [38] adjustment for simultaneous inference. However, comparison to the Tennessee unexposed group showed 7 physiological test differences: balance measured with eyes open and with eyes closed for 2 abnormalities, simple and choice reaction time 2 abnormalities, color discrimination errors 1 abnormality, and visual field performance 2 abnormalities. For the psychological tests: digit symbol substitution, vocabulary, verbal recall (immediate and delayed), and picture completion were significantly different. Testing for simultaneous inference reduced the significant differences for both sets of comparisons, but choice reaction time, balance and color discriminating errors, visual field performance, digit symbol substitution, vocabulary, visual recall, immediate and delayed verbal recall, and picture completion remained different compared to TN unexposed, only simple reaction time was dropped. Ohio near exposed had 7 differences from TN unexposed that were for color discrimination errors, visual field performance, immediate and delayed verbal recall, and picture completion.

Profile of Mood States mean scores were elevated at 53.1 in the 25 near exposed people versus 5.6 in the 22 distant exposed, people versus 5.6 in the 22 distant exposed, and 22.1 in the Tennessee unexposed people (*P* < 0.0001) (Table 2). Total abnormalities were correlated with symptom frequencies but not Profile of Mood States scores by regression analysis (*P* > .007) with 27.4% of the variance (*r* ^2^) explained.

More of the near exposed than for exposed or unexposed groups had ever smoked cigarettes: 40% versus 28%, but similar proportions, 16% versus 13% continued to smoke. Unexposed smokers from Tennessee were not different from nonsmokers for total neurobehavioral impairments. Regression of total neurobehavioral abnormalities against age, duration of smoking in years, and educational attainment showed only age was significant in the near exposed and no factor was significant in the far exposed (data are not shown).

The difference in total abnormalities, mean 4.3 ± 3.0, for the 25 near exposed compared to a mean of 2.5 ± 2.3 in the 22 distant exposed was statistically significant (by ANOVA, *P* < .011). The comparison of the near exposed group mean abnormalities of 4.3 to the Tennessee control's mean of 2.3 ± 2.1 was also statistically significant (*P* < .0001), as was the comparison of Ohio far-exposed people to Tennessee unexposed people that showed 6 differences and an abnormality score of 2.5 which was not significant (*P* < .879).

The near-exposed group had increased frequencies for shortness of breath when climbing stairs, but not at rest, or while walking nor when was wheezing more frequent. Their expiratory flows and vital capacities were significantly decreased (comparisons were adjusted for years of smoking) compared to the far exposed and to the unexposed (Table 3).

Frequencies of 18 of 35 symptoms were statistically significantly elevated in near exposed compared to far exposed (Table 4), and mean frequencies were ssd, 3.2 ± 1.7 in near exposed versus 1.9 ± 0.9 in far (*P* < .002) exposed and 2.6 ± 1.1 in the Tennessee unexposed (*P* < .038). Nine symptom frequencies were elevated compared to the Tennessee unexposed. Three general symptoms namely extreme fatigue, headache, and decreased smell joined eye irritation, loss of balance, loss of concentration, and losses of recent and long term memory. The near to farther away neighbor comparison added 6 chest symptoms: chest tightness, palpitation, shortness of breath, dry cough, dry mouth, throat tightness. This comparison added dizziness and lightheadedness to the balance category and somnolence, irritability and unstable mood. There were no differences between the exposed and unexposed groups for rheumatic or for lupus erythematosus complaints or for neurological diseases and psychiatric illnesses. No subject had substance dependency. The unexposed and near exposed and far exposed groups' did not differ in their occasional exposures to 15 occupations and groups of chemicals.

**Figure 1:**
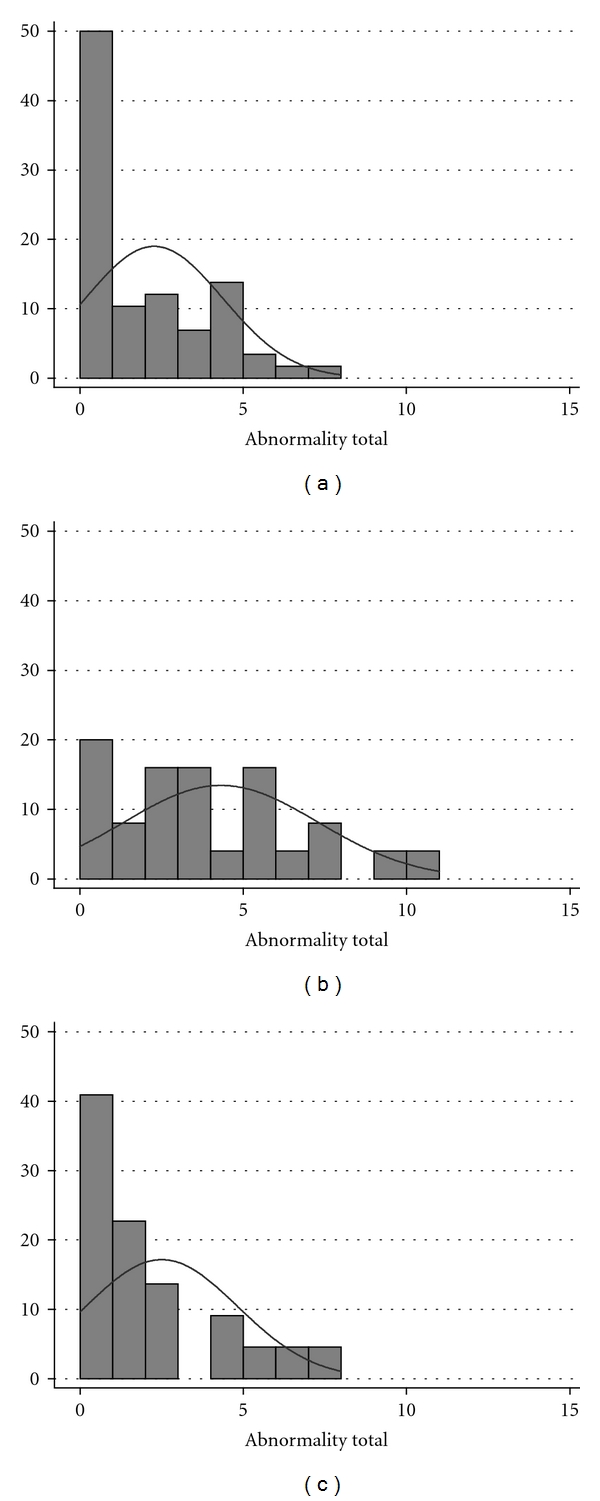
(a) Individual abnormality frequencies in regional Tennessee controls show a mean of 2.3 and a distribution skewed leftward. (b) The 25 hog-farm-exposed people had a symmetrical distribution of abnormalities with a mean frequency of 4.3. (c) The 22 Ohio hog-farm-distant-exposed people also had a skewed distribution of abnormalities with a mean frequency of 2.5.

The 58 Tennessee unexposed people's individual abnormality scores averaged 2.3 with distributions skewed to the left as plotted in Figure 1(a). The comparative abnormality scores for the 25 near exposed (mean 4.3) and 22 far exposed (mean 2.5) are plotted in Figures 1(b) and 1(c). The distribution of abnormalities of the Tennessee controls was skewed, increasing sharply from many with zero to one or two going to eight abnormalities. In contrast, the 25 near-exposed subjects had a symmetrical distribution around the mean of 4.3. The 22 Ohio distant-exposed subjects also had a symmetrical distribution of abnormalities around a mean of 2.5.

## 4. Discussion

The number of neurobehavioral impairments in people exposed around lagoons emitting hydrogen sulfide differed significantly from local more distant people and differed greatly from unexposed people in a nearby state. Significantly, lower expiratory flows indicated pulmonary impairment. Spot-check sampling in May 2003 and February 2005 shows H_2_S odors were mainly from several hog lagoons. Average indoor air hydrogen sulfide concentrations ranged from 0 to 30 ppb. Outdoor samples peaked at 1,600 ppb and indoor at 2,100 ppb with tap water running. Nevertheless, neurobehavioral impairments in these people were consistent with those from other hydrogen sulfide exposures, where levels were 1 to 5 ppm with peaks up to 100-fold higher [5, 10, 11]. Although distances to homes of impaired subjects from hog confinement, as a surrogate for hydrogen sulfide dose and total neurobehavioral abnormalities did not correlate, neither peak concentrations nor cumulative exposures were characterized and prevailing wind and humidity were assayed only on the days of sampling not for period of each season that would be needed to characterize doses.

There were significantly more abnormal tests in Paulding people near exposed and distant exposed than in regional Tennessee [13] referents and other unexposed (control) groups [7, 8, 39, 40]. These abnormalities are attributed primarily to hydrogen sulfide and other effluents from hog manure lagoons making it reasonably probable that local controls were significantly more abnormal than were regional referents, which suggests that both near and distant local groups shared exposures, probably to H_2_S. Although the inverse square of the distance from sources did not correlate with abnormalities. Among possible explanations are (1) inhaling a few breaths of a spike of H_2_S of 200 or more ppm can greatly impair human brain function and (2) the dynamic nature of this heavier-than-air gas movement that spread concentrations irregularly dependant on wind, protection, and depressions in the ground or buildings. Also, consider that hydrogen sulfide is attached to particles and particles contain Gram-negative bacterial endotoxin to cause the measured impairments.

Other possibilities such as considering that Ohio far-exposed people were biased for abnormality are inconsistent because fewer subjects were abnormal for tests of: blink reflex latency, peg placement, trail making, information, and similarities. Sectional differences in unexposed peoples function or impairment in the United States has not been found [39]. No adverse demographic, geographic, or other factors were found. If hydrogen sulfide is not the factor affecting both groups, we hypothesize a parallel (chemical) exposure, a shared Ohio factor. This problem has been encountered before [40]. No exposures to chlorinated solvents were found in reports of Environmental Protection Agency's monitoring of community culinary water. Other possible Ohio factors include atrazine, a herbicide widely used on corn fields, phosphorothioic acid (Famphur), an organophosphate insecticide used on corn and animals, and Gram-negative bacterial endotoxin that has been measured in hog confinement workers [41].

A review of 2,786 workers in swine confinement buildings from 14 studies [42] showed elevated frequencies of chronic cough, phlegm, chest tightness, wheezing, and acute intermittent symptoms. Respiratory symptoms were accompanied by decrements in flow and further drops during work. Chronic fatigue, muscle and joint pains, and dizziness were also described [42]. A later study of 54 male workers correlated reduced forced vital capacities to increased endotoxin levels in dust (mean 11,443 endotoxin units) by regression analysis [41].

Objectionable odor has been associated with elevated scores on the 5 adverse moods of the Profile of Mood States (POMSs) thus increasing the total Profile of Mood States score in 44 people living nearby indoor hog operation compared to age- and sex-matched control people [43]. The stench of these operations has made national news repeatedly [44, 45].

Interviews of 55 people living near confined animal feeding operations in North Carolina found increased headaches, burning eyes, running nose, sore throat, excessive coughing, and diarrhea compared to 50 more distant neighbors [46]. These observations increase health concerns about exposure to confined animal feeding operations [47] particularly as to whether there are measurable effects on functions related to these exposures. Such concern is irrespective of whether our exposed and control people are considered as one group varying in proximity to hydrogen sulfide sources or sharing an additional and as yet unknown toxic exposure.

The demonstration of neurobehavioral impairment from proximity to confined animal feeding operations in Ohio neighbors of hog raising indoors, CAFOs, adds abnormal functions to excessive symptoms that were quantified earlier. We also confirmed in downwind neighbors of hog confinement the reductions in vital capacity and flows found in hog confinement workers [41].

### 4.1. Limitations and Alternate Explanations

The results agree with those from occupational groups [3, 4] and environmental exposures [7, 8, 10]. Some people who lived on farms nearby may have had occupational exposure to hydrogen sulfide but this is unlikely as most farms had no meat or dairy animals. Increased complaints from being in an exposed group [48] do not impair performance on our neurobehavioral tests [9, 40]. Conscious manipulation is impossible for blink reflex latency, a test that can be done on unconscious subjects. In contrast, a subject could deliberately slow peg placement and trail making, but test givers recognize and correct such slowing. Also, such manipulation is unlikely because local near-exposed and distant-exposed groups performed equally to Tennessee unexposed. For balance and reaction time, the best of several trials was the score. Finally, intentional poor performance is unlikely because we asked experienced testers' to fake impairment on these tests and they could not do so. It would be even less likely for people naïve to these tests to coordinate “a group effort to affect scores.” The methods for visual field mapping were comparable for Ohio people without differences between exposed and controls. The systematic difference from Tennessee controls was due to a different method for the fields. Color testing was done on right and left eyes, the scores compared and consistent results were accepted. An explanation for variable results was sought in history and interview.

Information, picture completion, and similarity scores from the well-learned cultural domain were correlated with the highest grade attained in school as shown by others [32]. Inebriation had no role as no alcohol levels in expired breath were elevated. Cigarette smoking that raised carbon monoxide levels in alveolar air from 5 to 30 ppm has no adverse neurobehavioral effects [13, 49]. The reverse is true, chronic smoking of cigarettes has improved the speed of choice reaction time and peg placement (personal observation) and nicotine improves mental and physical functions [50, 51]. Decreased respiratory flows after adjusting for effects of smoking appeared due to hydrogen sulfide, as observed previously [10, 11].

After sewer workers died from inhalation of gas in Paris and London in the mid 1800s, Christison [1] attributed the deaths to sulfurated hydrogen, now known as hydrogen sulfide. Poisonings of bystanders are still reported regularly from hydrogen sulfide escaping from geothermal sites, refineries, desulfurization plants, pipelines, hog husbandry buildings, waste lagoons, cattle feed lots, dairy buildings, wood pulping lagoons, and so forth [5, 6, 10]. Occupational exposures to hydrogen sulfide include shale oil [52], ocean fishing [53], oil refining [54, 55], and cleaning geothermal (hot) springs [56].

### 4.2. Mechanisms of Toxicity

Hydrogen sulfide poisons the brain and mitochondria by irreversibly combining with iron in respiratory enzymes, cytochrome oxidases, thus stopping oxidative phosphorylation. It stimulates the respiratory center, increasing hydrogen sulfide intake for a breath or two [57, 58]. Lower doses increase brain neuromediators by inhibiting monoamine oxidase [58] and there is evidence that hydrogen sulfide is the brain's third gaseous mediator [59].

Sensitive testing showed permanent brain dysfunction in workers thought to have recovered from hydrogen sulfide exposures [52, 54, 55]. They had cognitive and recall memory deficits reduced problem solving ability, impaired balance, slowed reaction time, and scotomata, losses in their visual fields [11, 25, 57]. The present observations from human exposure to hydrogen sulfide at the lower end of concentrations expected to produce adverse effects are tentative. They replicate earlier studies and invite validation by others. Unfortunately, it appears that studies of groups of people exposed environmentally in incidents associated with symptoms will have similar limitations in dose estimation as did this study.

Data are insufficient to propose a safe dose of hydrogen sulfide. This is because single brief exposures to sublethal doses may severely impair brain function and so does years of exposure to levels below 1 ppm. A dose-time relationship has not been found [11, 53]. As a safe level cannot be proposed, it would be prudent to separate people from all sources of hydrogen sulfide: feed lots, tanneries, oil refineries, and natural gas processing (desulphurization), and ponds and lagoons contain sulfur that becomes anaerobic as in geothermal sites such as hot springs. Information from many sources suggests that proximity is the important and geothermal factor in toxicity despite effects of other gases, wind velocity and direction, land contour, and temperature.

The precautionary principle recommends that odors, perceived at levels of H_2_S above 30 ppb, are the cue to escape further exposure. Delay may let olfactory fatigue abolish the warning and invite damage.

## Figures and Tables

**Table 1:** 25 people near hog lagoons compared to 22 distant exposed and to 58 Tennessee unexposed (compared as means of percent predicted values, by analysis of variance, ANOVA).

Percent predicted	A: distant exposed 22 mean ±sd	B: near exposed 25 mean ± sd	A versus B *P* value	C: unexposed TN 58 mean ± sd	B versus C *P* value (Holm p)	A versus C *P* value (Holm p)
Age (years)	56.6 ± 16.0	50.4 ± 16.8	.284	56.8 ± 18.1	.0001	.977
Educational level (years)	12.1 ± 1.7	13.0 ± 2.2	.195	12.1 ± 1.2	.817	.236
Simple reaction time	100.9 ± 4.2	101.3 ± 3.4	.757	103.1 ± 5.3	.017* .24^+^	.228
Choice reaction time	101.2 ± 3.0	101.5 ± 2.9	.824	103.3 ± 4.0	.0005* .01^+^	.113
Balance sway speed						
Eyes open	104.6 ± 2.6	136.4 ± 29.5	.0014*	106.9 ± 34.4	.0002* .004^+^	.831
Eyes closed	109.7 ± 26.7	149.3 ± 43.5	.003	95.1 ± 24.5	.0016* .03^+^	.147
Blink reflex latency R-1						
Right	90.4 ± 7.3	101.2 ± 13.0	.010	96.6 ± 15.4	.745	.473
Left	91.8 ± 10.3	92.7 ± 12.5	.861	105.2 ± 16.7	.963	.019*
Color discrimination errors						
Right	63.9 ± 47.0	42.3 ± 39.2	.173	66.7 ± 52.5	.0002* .004^+^	.883^+^
Left	56.2 ± 35.5	48.8 ± 41.8	.583	44.3 ± 38.3	.0001* .0027^+^	.394
Visual field performance						
Right	121.7 ± 14.8	110.1 ± 14.5	.033	119.9 ± 8.0	.0001* .003^+^	.0001* .0028^+^
Left	122.9 ± 21.3	110.6 ± 14.2	.070	124.1 ± 10.6	.0001* .0032^+^	.0001* .003^+^
Grip strength						
Right	105.7 ± 17.8	99.0 ± 18.9	.264	93.7 ± 14.5	.733	.040*
Left	102.1 ± 17.2	95.4 ± 22.1	.336	90.9 ± 12.4	.784	.070
Cognition						
Culture fair	107.1 ± 27.9	101.4 ± 15.9	.493	97.6 ± 23.2	.842	.348
Digit symbol	101.3 ± 12.6	88.2 ± 20.5	.030* .60^+^	91.4 ± 18.0	.0001* .0024^+^	.089
Vocabulary	89.9 ± 34.9	66.5 ± 27.2	.046	72.5 ± 34.1	.002* .03^+^	.197
Verbal recall						
Immediate	84.8 ± 27.5	78.5 ± 23.3	.488	75.3 ± 17.4	.0008* .015^+^	.309
Delayed	57.9 ± 33.5	65.1 ± 35.1	.555	38.9 ± 29.5	.0001* .0023^+^	.131
Pegboard	117.7 ± 20.3	102.2 ± 23.2	.050	108.9 ± 15.3	.769	.221
Trails A	99.9 ± 7.0	101.4 ± 5.6	.512	106.5 ± 10.0	.149	.044
Trails B	100.5 ± 7.9	103.7 ± 7.6	.238	96.3 ± 16.8	.423	.372
FTNWE right	94.4 ± 8.5	98.6 ± 7.4	.151	97.1 ± 8.4	.249	.395
FTNWE left	96.5 ± 8.9	102.6 ± 8.1	.054	99.6 ± 12.3	.442	.428
Information	98.4 ± 39.5	85.4 ± 31.9	.318	94.3 ± 36.4	.403	.780
Picture Completion	69.7 ± 36.1	69.7 ± 42.4	.998	58.8 ± 40.4	.0009* .016^+^	.450
Similarities	102.6 ± 30.5	86.5 ± 41.5	.209	95.3 ± 47.5	.650	.608

Total Abnormalities	2.5 ± 2.3	4.3 ± 3.0	.011	2.3 ± 2.1	.001	.879

*: P < 0.05.

*^+^:* *P* < 0.05 after Holm's correction for multiple inference [38].

**Table 2:** Profile of mood states (POMSs) for 25 near exposed compared to 22 distant exposed in Paulding, Ohio, and 58 unexposed Tennessee subjects (compared as means of percent predicted values, by analysis of variance, ANOVA).

POMS	A: 22 distant exposed mean ± sd	B: 25 near distant exposed mean ± sd	A versus B *P* values	C: 58 TN unexposed mean ± sd	A versus C *P* values
Score*	5.6 ± 18.6	53.1 ± 45.5	.0001	22.1 ± 25.0	.0001
Range	−28 to 46	−11 to 164	**—**	5 to 130	**—**
Tension	7.2 ± 4.0	14.9 ± 7.7	.0001	8.9 ± 4.6	.0001
Depression	4.1 ± 4.1	14.7 ± 12.1	.0003	7.9 ± 7.1	.002
Anger	4.8 ± 4.5	15.4 ± 11.9	.0003	7.7 ± 6.5	.0003
Vigor	19.8 ± 7.0	14.7 ± 7.2	.018	17.0 ± 6.2	.137
Fatigue	5.5 ± 3.4	12.2 ± 7.6	.0004	8.3 ± 5.6	.01
Confusion	3.8 ± 2.5	10.6 ± 5.8	.0001	6.4 ± 3.7	.0001

* Vigor subtracts from the sum of affective states, so can be minus 28.

**Table 3:** Pulmonary function tests in 25 near-exposed subjects compared to 22 distant exposed in Paulding, Ohio, and 58 unexposed Tennessee subjects (compared as means of percent predicted values, by analysis, of variance, ANOVA).

	A: 22 distant exposed Mean ± sd	B: 25 near exposed Mean ± sd	A versus B *P* values	C: 58 TN unexposed Mean ± sd	B versus C *P* values	A versus C *P* values
FVC	97.0 ± 12.8	87.6 ± 10.7	.014*	101.6 ± 15.2	.0001*	.180
FEV_1_	94.5 ± 11.7	85.5 ± 15.4	.028*	93.6 ± 15.2	.025*	.070
FEF_25-75_	98.4 ± 20.0	91.9 ± 30.8	.223	88.1 ± 35.0	.633	.070
FEF_75-85_	90.8 ± 24.2	96.4 ± 54.1	.080	78.1 ± 52.7	.133	.191
FEV_1_/FVC	77.9 ± 3.3	76.1 ± 7.3	.301	72.8 ± 9.5	.130	.014*

* Statistically significant values.

**Table 4:** Symptom frequencies (1 to 11 scale) for 25 near-exposed subjects compared to 22 distant-exposed and 58 unexposed subjects.

Symptom	A: 25 near exposed	B: 22 distant exposed	A versus B *P* values	C: 58 TN unexposed	A versus C *P* values
Skin irritation	4.1 ± 3.5	2.5 ± 2.1	.071	3.2 ± 2.5	.222
Deformed finger nails	1.4 ± 1.3	1.4 ± 1.2	.893	1.5 ± 1.3	.655
Chest tightness	2.6 ± 2.5	1.5 ± 0.8	.049*	2.0 ± 1.5	.234
Palpitations	2.0 ± 1.5	1.6 ± 1.7	.034*	2.3 ± 2.1	.580
Burning-tightness of chest	2.0 ± 1.9	1.4 ± 0.7	.170	1.9 ± 1.6	.977
Shortness of breath	2.7 ± 2.2	1.5 ± 1.1	.035*	3.1 ± 2.0	.418
Dry cough	3.0 ± 2.2	1.9 ± 1.0	.040*	2.5 ± 1.8	.362
Cough with mucus	2.9 ± 2.4	2.0 ± 1.3	.127	2.6 ± 1.9	.521
Cough with blood	1.4 ± 1.2	1.0 ± 0.0	.133	1.1 ± 0.6	.195
Dry mouth	4.1 ± 3.0	2.0 ± 0.9	.002*	3.1 ± 2.5	1.25
Throat tight	3.8 ± 2.9	2.3 ± 1.5	.035*	2.8 ± 1.9	.091
Eye irritation	3.8 ± 2.9	2.1 ± 1.6	.015*	2.4 ± 2.1	.013*
Decreased smell	3.4 ± 3.1	2.1 ± 2.2	.095	2.1 ± 2.0	.021*
Headache	5.7 ± 3.5	3.2 ± 2.4	.007*	4.1 ± 2.4	.017*
Nausea	2.2 ± 1.6	1.8 ± 1.5	.313	2.5 ± 1.7	.575
Dizziness	3.0 ± 2.7	1.5 ± 0.9	.017*	2.1 ± 1.6	.059
Lightheadedness	2.8 ± 1.9	1.7 ± 0.8	.019*	2.5 ± 1.7	.486
Exhilaration (unusual)	1.2 ± 1.6	1.1 ± 0.5	.704	1.8 ± 1.8	.062
Loss of balance	3.3 ± 2.6	1.5 ± 0.9	.003*	1.9 ± 1.2	.001*
Loss of consciousness	1.3 ± 1.1	1.0 ± 0.2	.243	1.2 ± 0.4	.356
Extreme fatigue	4.9 ± 3.4	1.8 ± 1.6	.0003*	3.1 ± 2.3	.005*
Somnolence	3.2 ± 2.9	1.3 ± 0.6	.004*	2.5 ± 2.2	.242
Insomnia	3.3 ± 3.1	2.3 ± 2.1	.204	2.6 ± 2.5	.291
Wake frequently	3.9 ± 3.1	2.5 ± 2.0	.089	2.7 ± 2.5	.014
Sleep few hours	3.7 ± 2.7	2.4 ± 2.4	.100	2.6 ± 2.5	.086
Irritability	4.5 ± 3.4	2.3 ± 1.7	.009*	3.7 ± 2.4	.236
Loss of concentration	4.6 ± 3.6	2.1 ± 1.7	.005*	3.2 ± 2.1	.034*
Loss of recent memory	5.8 ± 3.5	2.3 ± 1.6	.0001*	3.2 ± 2.6	.0002*
Long-term memory loss	4.1 ± 3.1	1.7 ± 1.3	.001*	2.4 ± 2.2	.006*
Unstable moods	3.6 ± 3.2	1.3 ± 0.5	.002*	2.4 ± 2.1	.064
Loss of libido	4.2 ± 3.2	2.7 ± 2.1	.060	3.8 ± 3.3	.537
Decreased alcohol tolerance	1.6 ± 1.2	1.6 ± 1.2	.992	2.2 ± 1.9	.165
Indigestion	3.2 ± 1.9	3.0 ± 2.2	.737	2.8 ± 2.2	.430
Loss of appetite	2.2 ± 1.7	1.5 ± 1.2	.121	2.4 ± 1.9	.570
Swollen stomach	3.0 ± 3.0	2.0 ± 2.0	.177	2.8 ± 2.5	.759
*Tingling navel*	1.0 ± 0.2	1.0 ± 0.2	.928	1.2 ± 0.8	.404
*Itching gums*	1.0 ± 0.0	1.0 ± 0.0	1.00	1.2 ± 0.5	.068

Symptom frequency mean	3.2 ± 1.70	1.9 ± .91	.002	2.6 ± 1.1	.038*
Symptom frequency range	1.22 to 6.57	1.11 to 5.11		1.18 to 5.68	

* Statistically significant values.
